# The Versatile Roles of Sulfur-Containing Biomolecules in Plant Defense—A Road to Disease Resistance

**DOI:** 10.3390/plants9121705

**Published:** 2020-12-03

**Authors:** András Künstler, Gábor Gullner, Attila L. Ádám, Judit Kolozsváriné Nagy, Lóránt Király

**Affiliations:** Plant Protection Institute, Centre for Agricultural Research, 15 Herman Ottó Str., H-1022 Budapest, Hungary; kunstler.andras@atk.hu (A.K.); gullner.gabor@atk.hu (G.G.); adam.attila@atk.hu (A.L.Á.); nagy.judit@atk.hu (J.K.N.)

**Keywords:** cysteine, defensin, glucosinolate, glutathione, hydrogen peroxide, hydrogen sulfide, reactive sulfur species, salicylic acid, sulfur-containing defense compounds, thionin

## Abstract

Sulfur (S) is an essential plant macronutrient and the pivotal role of sulfur compounds in plant disease resistance has become obvious in recent decades. This review attempts to recapitulate results on the various functions of sulfur-containing defense compounds (SDCs) in plant defense responses to pathogens. These compounds include sulfur containing amino acids such as cysteine and methionine, the tripeptide glutathione, thionins and defensins, glucosinolates and phytoalexins and, last but not least, reactive sulfur species and hydrogen sulfide. SDCs play versatile roles both in pathogen perception and initiating signal transduction pathways that are interconnected with various defense processes regulated by plant hormones (salicylic acid, jasmonic acid and ethylene) and reactive oxygen species (ROS). Importantly, ROS-mediated reversible oxidation of cysteine residues on plant proteins have profound effects on protein functions like signal transduction of plant defense responses during pathogen infections. Indeed, the multifaceted plant defense responses initiated by SDCs should provide novel tools for plant breeding to endow crops with efficient defense responses to invading pathogens.

## 1. Introduction

The role of sulfur in the resistance of crops against fungal diseases became obvious at the end of the 1980s when atmospheric sulfur depositions were so much reduced by clean air acts that sulfur deficiency became a widespread nutrient disorder in Western European agriculture and the infection of crops with certain diseases became increasingly obvious, mostly in Scotland and Germany [1]. The emission of sulfur oxides into the atmosphere was also dramatically reduced in Central Europe at the end of the last century, mainly due to modernization of thermal power stations and to the reduction in fossil fuel combustion. At the beginning of this century, the level of emission of different sulfur oxides (ingredients of acid rain) was reduced by more than 70% as compared to emissions in 1980 [2]. The reduction in anthropogenic sulfur deposition resulted in progressive sulfur deficiency in plant mineral nutrition. Therefore, sulfate salts were applied to fields to cover the sulfur demand of plants. Interestingly, such agricultural field experiments showed that soil-applied sulfur in the form of inorganic sulfate salts can markedly increase the disease resistance of crops against certain fungal pathogens. A significant repressive effect of soil-applied sulfur on the infection of oilseed rape with *Pyrenopeziza brassicae*, grapes with *Uncinula necator*, and potato tubers with *Rhizoctonia solani* was found [3,4,5]. These results led to the development of the concept of sulfur-induced resistance (SIR) [1,4,6,7]. This new disease resistance form has also been observed in pathophysiological and biochemical experiments using plants grown under controlled greenhouse conditions, when this phenomenon was described as sulfur-enhanced defense (SED) [5,8]. The concepts of SIR and SED describe the same phenomenon from different experimental approaches, from an agricultural and a plant biological point of view, respectively. In spite of numerous studies, the mechanisms underlying SIR/SED are, however, far from understood.

**Figure 1:**
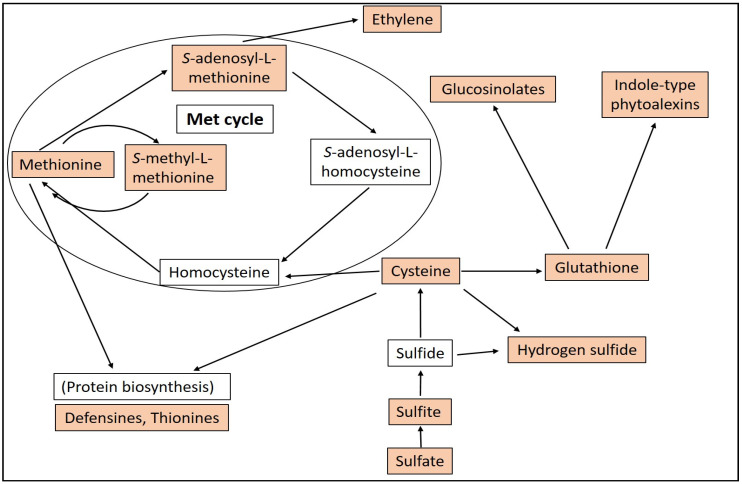
Schematic representation of biosynthetic pathways of the most important sulfur-associated compounds in plants. Sulfur-associated compounds mentioned in this review are highlighted.

Acclimation and adaptation processes are crucial for plants to survive in changing environments and the goal for the plant is to optimize the use of available sulfur to match the demand for growth and development, and resistance to biotic and abiotic stress [9]. Sulfur requirements can vary among plant families. Members of the *Brassicaceae* are found to be the most sulfur-dependent group of plants, followed by *Fabaceae* and *Poaceae* [10]. The primary sulfur source of the plants are inorganic sulfate anions available from the soil [11]. The sulfate anion is taken up from the soil by specialized sulfate transporter proteins, which are localized in the epidermal cells of the roots [12]. Excess sulfate is transported to the leaves and is stored in vacuoles that constitute a large S reservoir for plant metabolism [13]. The transportation of sulfate within or between plant cells is also mediated by sulfate transporters [14]. Sulfate in plant cells is activated to form adenosine 5′-phosphosulfate, a process catalyzed by ATP sulfurylase [15]. The activated sulfate is reduced in a multistep pathway in which eight electrons are added to form sulfide through sulfite as an intermediate form [16]. Sulfide, together with *O*-acetylseryne (OAS), forms cysteine (Cys), a reaction catalyzed by two enzymes, serine acetyltransferase (SAT) and *O*-acetylserine(thiol)lyase (OASTL) [17]. In these processes the sulfur atom is ultimately incorporated into Cys, the first organic molecule carrying reduced sulfur and a central hub of SDC biosynthesis in plants [18,19,20,21] (Figure 1).

Because of the importance of sulfur-containing defense compounds (SDCs) for plants, sulfate assimilation and its transformation to SDCs is tightly regulated. Generally, the pathway is regulated by demand, namely it is repressed when reduced sulfur is available and activated by high demand for reduced sulfur [22]. Furthermore, sulfate assimilation in plants is interconnected with the assimilation of nitrate and carbon [9,23,24]. A transcription factor, sulfur limitation 1 (SLIM1) has been identified in *Arabidospsis* that regulates the main pathways of sulfate uptake and metabolism in sulfate deficient plants by upregulating the expression of different sulfate transporters especially SULTR1;2 which is the major sulfate uptake facilitator in *Arabidopsis* [25]. Moreover, SLIM1 affects genes involved in the degradation of glucosinolates (GSLs) as well [25]. Furthermore, in Cys biosynthesis the limiting enzyme of the pathway is SAT. Different isoforms of SAT in various species and plant organelles display varying degrees of feedback inhibition by cysteine [26]. In addition, levels of OAS in plants are rapidly altered during S deficiency and tightly correlated with regulators of sulfur metabolism, that have key roles in balancing plant sulfur pools, including *gamma-glutamyl cyclotransferase 2;1* (*GGCT2;1*) *sulfur deficiency induced genes* (*SDI1* and *SDI2*) and *more sulfur accumulation1* (*MSA1*) [10]. GGCT2;1 degrades the glutathione (GSH) pool to its amino acid constituents, glutamate, Cys and glycine, possibly to mobilize Cys under sulfate shortage conditions when de novo Cys synthesis is limited [27]. SDI1 and SDI2 are identified as repressors of GSLs via direct interaction with the transcription factor MYB28 repressing the transcription of GSL biosynthetic genes in sulfur deficient plants [28]. MSA1 modulates *S*-adenosyl-l-methionine (SAM) biosynthesis and DNA methylation affecting genes connected with sulfate uptake (SULTR1;2) and GSL regulation [29]. In plants, Cys is the metabolic hub that integrates the products of reductive assimilation of sulfate, nitrate, and CO_2_. In particular, sulfate assimilation is mediated by the sensor kinase target of rapamycin (TOR) that does not directly sense Cys but rather the supply of its precursors [23]. In summary, this mechanism allows plants to coordinate the fluxes of carbon, nitrogen, and sulfur for efficient Cys and SDC biosynthesis under varying external nutrient supply. Finally, the signaling pathways of different phytohormones are linked to efficient S use in plant defense pathways and plant developmental processes and metabolism under both normal and stress conditions (see [9] and references within).

Cytosolic Cys homeostasis is essential in plant immunity [21]. The central role of Cys is to serve as the precursor of a wide variety of antimicrobial or antioxidative thiol compounds such as GSH, thionins, defensins, phytoalexins, glucosinolates and S-containing volatiles [7,30,31,32]. In addition, cysteine residues in proteins often participate in the redox regulation of protein functions through the formation or reduction in disulfide bridges [33,34]. The biosynthesis of sulfur-containing defense compounds is hormonally regulated [30]. Particularly, jasmonic acid plays an important role in the activation of the sulfate reduction pathway that precedes synthesis of SDCs [35]. The role of different SDCs in plant disease resistance has been intensively investigated in recent years [7,8,9,36,37]. This review attempts to recapitulate the possible roles of sulfur-containing plant metabolites in the resistance of plants to pathogen infections.

## 2. Sulfur Containing Amino Acids (SAAs) in Plant Disease Resistance

### 2.1. Cysteine

Cysteine (Cys) is the final product of sulfur assimilation and the first organic compound containing reduced sulfur synthesized by plants [17]. The central role of Cys in plants is defined as being a sulfur containing amino acid in proteins and as a precursor for a large number of important sulfur containing biomolecules, which have major roles in plant disease resistance (Figure 1). However, Cys is not only a precursor compound but also a major player in the regulation of plant defense responses. It has been demonstrated that two enzymes involved in Cys biosynthesis and degradation, respectively, have a huge impact on disease resistance of *Arabidopsis thaliana* to the hemibiotrophic *Pseudomonas syringae* pv. *tomato* (*Pst*) DC3000 and the necrotrophic *Botrytis cinerea* [18]. The enzyme *O*-acetylserine(thiol)lyase (OASTL) combines a sulfide molecule with *O*-acetylserine, which is the final step of cysteine biosynthesis. OASTL-deficient mutant plants showed reduced Cys and GSH levels and increased susceptibility to both pathogens. On the other hand, l-cysteine desulfhydrase (DES1) degrades Cys in the plant cytosol, accordingly, *DES1* mutants displayed increased Cys and GSH contents and lower pathogen levels [18]. Furthermore, these authors demonstrated that cytosolic Cys homeostasis is essential for the initiation of the hypersensitive response (localized host necrosis, HR) during effector triggered immunity (ETI) to *Pst* DC3000 *avrRpm1* [18]. Others have found that *Arabidopsis* ONSET OF LEAF DEATH3 (*old3-2*) mutants are lacking functional OASTL-A1 in the cytosol and these plants also show increased susceptibility to *Pst* DC3000 [38].

The first line of plant defense comprises pathogen recognition initiated by different plant receptors localized on the surface or inside of plant cells [39]. For example, Cys-rich receptor-like kinases (CRKs) in *A. thaliana* are up-regulated when plants are treated with bacterial flagellin flg22. The silencing of genes encoding bacterial flagellin-inducible CRKs leads to enhanced susceptibility to *Pst* DC3000, while overexpression of *CRK28* in *Arabidopsis* increased disease resistance to this bacterial pathogen [40]. To understand the role of CRK28 in disease resistance, the gene was also overexpressed in *Nicotiana benthamiana.* Pathogen perception of *N. benthamiana* induced an extracellular burst of reactive oxygen species (ROS), and the resulting oxidative stress facilitated the formation of multiple intra and intermolecular disulfide bonds between the eight extracellular Cys residues of *CRK28*. Mutating four extracellular Cys to alanine (Ala) completely abolished the four disulfide bounds within CRK28 and disrupted CRK28-mediated cell death during pathogen infection leading to the suppression of plant defense responses [40]. A similar phenomenon was observed in a resistant wheat cultivar infected with leaf rust (*Puccinia triticina*). A novel wheat cysteine-rich receptor-like kinase gene, *TaCRK2*, was identified that is specifically induced in this incompatible interaction. Knockdown of *TaCRK2* by *Barley stripe mosaic virus*-induced gene silencing leads to a dramatic increase in the HR area and the number of haustorial mother cells at infection sites, indicating a suppressed resistance [41]. It has also been shown by these authors that the *TaCRK2* receptor is localized in the endoplasmic reticulum [41]. Hydrogen peroxide (H_2_O_2_) is a major ROS produced in plants extracellularly in response to external stresses such as pathogen infection [42]. It has been reported recently that a novel leucine-rich-repeat receptor kinase, hydrogen-peroxide-induced Ca^2+^ increase (HPCA1), is the first extracellular H_2_O_2_ receptor identified in plants [43]. HPCA1 is localized in the *A. thaliana* plasma membrane and Cys residues are located at the HPCA1 extracellular domain. In the presence of H_2_O_2_, Cys-SH residues are activated via covalent modification, resulting in disulfide bridges. This leads to autophosphorylation of HPCA1 that mediates H_2_O_2_-induced activation of Ca^2+^ channels in guard cells which is required for stomatal closure [43], e.g., during resistance to bacterial infections.

It is worth mentioning that Cys also has direct antifungal effects. Cysteine inhibited both spore germination and mycelial growth in a concentration-dependent manner of the fungal pathogens *Phaeomoniella chlamydospora* and *Phaeoacremonium minimum*, which cause the grapevine trunk (esca) disease [44]. Using ^35^S-cysteine, it was demonstrated that the amino acid was absorbed following leaf spraying and transported to the trunk, which is the area where the fungal pathogens are localized in the course of the development of esca disease [44]. Similar antifungal effects of Cys were also shown for other fungal pathogens such as *B. cinerea* [45] and *Eutypa lata* [46]. In fact, Cys can display toxic properties in plants, including irreversible thiol oxidation, formation of hydroxyl radicals (OH^•^) and hydrogen sulfide (H_2_S), which are presumably related to its antifungal effects [32,47].

### 2.2. Methionine

The other important SAA in plants is methionine (Met), playing a central role in cellular metabolism, including protein synthesis, reactions of transmethylation through *S*-adenosyl-l-methionine (SAM) [48], as well as different defense reactions to biotic stresses. For example, the disease severity caused by *Sclerospora graminicola* infection was drastically reduced in a susceptible cultivar of pearl millet (*Pennisetum glaucum*) treated with Met [49]. Met treatment induces generation of hydrogen peroxide (H_2_O_2_), a key element in plant defense signaling, and upregulates the expression of different defense-related genes in grapevine (*Vitis vinifera*) [50]. Met treatment also reduced *Plasmopara viticola* development in grapevine plants. Furthermore, it was observed that Met possesses direct antifungal activity, however, this was moderate as compared to Cys under in vitro and in vivo conditions [50]. A Met derivative, *S*-methylmethionine (SMM) is a non-protein amino acid occuring naturally in plants. It has been demonstrated that SMM pretreatments maintain normal plant physiology by guarding and upholding the photosynthetic activity in *Maize dwarf mosaic virus* (MDMV) infected maize, however, the virus levels remain unchanged [51]. On the other hand, pretreatments with *S*-methylmethionine-salicylate (MMS), an artificial compound synthetized from SMM and salicylic acid (SA), successfully contribute to decreasing both the RNA and coat protein contents of MDMV in infected maize [52].

Potyviral helper component proteinase (HCPro) of *Potato virus A* (PVA) is a well-characterized pathogenicity factor causing a suppression of antiviral RNA silencing. It has been shown that HCPro may suppress antiviral RNA silencing in *N. benthamiana* through local disruption of the methionine cycle. The methionine cycle is using Met to supply *S*-adenosyl-l-methionine (SAM) to various in planta methylation processes. In this reaction cycle, *S*-adenosyl-l-homocysteine (SAH) is produced from SAM and SAH is further converted to homocysteine and then back to Met (Figure 1). HCPro acts together with other viral and host proteins to locally inhibit *S*-adenosyl-l-methionine synthase (SAMS) and *S*-adenosyl-l-homocysteine hydrolase (SAHH), which are the key enzymes of the Met cycle. This leads to the inhibition of small RNA methylation and destabilization of small interfering RNAs, resulting in suppression of RNA antiviral silencing and increased susceptibility to the potyvirus PVA [53]. Furthermore, in potex–potyviral synergisms, HCPro is known to enhance the pathogenicity of the potexvirus partner. A synergistic interaction of two plant viruses is typically manifested as severe symptoms and increased accumulation of both viruses in the host plant. In line with this, *Potato virus X* (PVX) accumulation in *N. benthamiana* is increased by the presence of PVA [54]. Interestingly, the same authors have also shown that silencing of SAHH (a key enzyme of the Met cycle) causes a similar increase in PVX accumulation. Furthermore, silencing of both Met cycle enzymes, SAHH and SAMS, also caused a significant reduction in GSH levels in PVX infected plants. The common precursor of both GSH and homocysteine, a central component of the Met cycle, is Cys. Therefore, the reduction in GSH levels could indicate the fact that when the Met cycle is disrupted during PVX infection, plant cells channel the Cys flux towards homocysteine rather than GSH biosynthesis. Importantly, knocking down the expression of GSH synthetase resulted in increased PVX accumulation pointing to the direct role of GSH in virus resistance [54]. Silencing Met cycle genes encoding SAHH and homocysteine methylase (MS) also leads to decreased resistance against *Ralstonia solanacearum* in tomato (*Solanum lycopersicum*) hosts [55]. During DNA de/methylation, plants reprogram their transcriptome and manage their genome stability to maximize their ability for adaptation of biotic (and abiotic) stresses such as pathogen infection [56]. It has been presented that a decrease in plant DNA methylation was accompanied by enhanced defense to *Blumeria graminis* f. sp. *tritici*, supporting a role of DNA de/methylation in *Aegilops tauschii* defense responses [57]. The role of DNA demethylation has been also demonstrated in disease resistance of *Arabidopsis* to *Pst* DC3000 infection. A loss-of-function mutation in the demethylase, repressor of silencing 1 (*ROS1*), enhances vascular spreading of a green fluorescent protein (GFP)-tagged *Pst* DC3000 in leaf secondary veins [58]. Furthermore, pathogenesis related gene 1 (*PR-1*) induction was reduced in *ros1* mutant plants treated with bacterial flagellin flg22, indicating that *ROS1* acts as a positive regulator of SA-dependent defense responses [58].

## 3. Glutathione (GSH) in Plant Disease Resistance

Glutathione (reduced form GSH; oxidized form GSSG) is the major non-protein thiol in plants [59]. It plays a role as a non-enzymatic antioxidant in the ascorbate-glutathione cycle, and participates in many detoxification reactions in plants [60,61,62]. Furthermore, GSH is also known as a central regulator of plant signaling during plant–pathogen interactions [63,64].

### 3.1. GSH Correlates with Plant Resistance

The positive correlation between GSH and disease resistance has been reported in several papers [54,60,65,66,67,68,69,70]. For example, it has been presented that a substantial increase in foliar GSH levels and an increase in the ratio of reduced to oxidized glutathione was detectable in two resistant oat lines (*Avenna sativa*) but not in a susceptible one 24 h after inoculation with *Blumeria graminis* f. sp. *avenae* [66]. The prominent role of glutathione in plant disease resistance is also underlined by the observation that the injection of the effector protein RipAY by the bacterium *Ralstonia solanacearum* into host plant cells correlates with GSH degradation [71]. RipAY has a ɣ-glutamyl cyclotransferase activity and the transient expression of RipAY in *N. benthamiana* greatly lowered GSH levels and suppressed plant immunity/disease resistance. Interestingly, bacterial cells have an excellent safety mechanism to prevent unwanted RipAY enzyme activity because RipAY is specifically activated only by plant thioredoxins but not by bacterial thioredoxins [71]. Although research results primarily support the pivotal role of GSH in plant disease resistance responses, there are cases where high GSH levels may be associated with susceptibility. For example, in barley (*Hordeum vulgare*) infected with its powdery mildew (*Blumeria graminis* f. sp. *hordei*), susceptible plants displayed a significant increase in total glutathione (GSH + GSSG) contents at 7 days after inoculation [72]. This is a later stage of pathogenesis when pathogen-induced visible symptoms (powdery mildew) develop and glutathione may contribute to a reducing environment required for a biotrophic pathogen. On the other hand, it is noteworthy to mention that glutathione was not assayed at early time points after inoculation, where it could potentially play a role in modulating/signaling resistance responses to powdery mildew [72]. Interestingly, however, it has been shown that in resistant soybeans GSH levels were low from the initial phases of nematode (*Heterodera glycines*) infection, as compared to a susceptible cultivar. In resistant soybeans low levels of GSH lead to increased H_2_O_2_ levels and reduced nematode accumulation. In contrast susceptible plants contain higher levels of GSH and lower H_2_O_2_. In the susceptible cultivar the reduction in GSH levels by l-buthionine-[S,R]-sulfoximine (BSO) increases H_2_O_2_ and the resistance to *H. glycines* [73].

### 3.2. Artificial Modification of GSH Levels in Plants Affects Disease Resistance

Artificially increasing GSH contents in plants induces disease resistance to different pathogens. Overexpression of SAT and OASTL (Cys biosynthesis) as well as gamma-glutamylcysteine synthetase (GSH1) (GSH biosynthesis) in *Nicotiana tabacum* led to increased levels of GSH associated with enhanced defense responses to Pst DC3000, *Botrytis cinerea* and *Tobacco mosaic virus* (TMV) [74,75,76]. Furthermore, transient elevation of GSH in tobacco by “GSH feeding” leads to enhanced PR-1a expression [77]. Infiltration of tobacco leaves with GSH two days before TMV inoculation successfully reduced TMV symptoms and virus levels in infiltrated leaves [76]. The application of the synthetic Cys precursor l-2-oxothiazolidine-4-carboxylic acid (OTC) elevated GSH contents in spinach cells [78] and Cys and GSH levels in maize [79]. As discussed above, high GSH contents correlate with resistance during different pathogen attacks. In line with these findings, OTC pretreatments markedly increased GSH levels in tobacco (*N. tabacum* cv. Xanthi), and additionally, OTC pretreatment resulted in both the reduction in disease symptoms and virus contents in TMV infected leaf discs [80]. A similar phenomenon was observed in *Zucchini yellow mosaic virus* (ZYMV) infected oil pumpkin (*Cucurbita pepo* subsp. *pepo* var. styriaca) plants. Treatment with OTC increased the levels of GSH inducing suppression, reduction, and delay of ZYMV symptoms and reduced virus accumulation during a compatible plant-virus interaction [81]. In *Plum pox virus* (PPV)-inoculated pea and peach plants, OTC treatments suppressed disease symptoms but PPV contents were not significantly reduced [82,83,84]. Injecting tobacco leaves with OTC increased GSH contents and plant resistance to TMV and the powdery mildew *Euoidium longipes* [76,85].

In contrast to physiological (optimal) GHS levels, GSH deficiency in plants generally leads to increased susceptibility to different pathogens. In this regard, it has been demonstrated that sufficient sulfate supply is an important component of plant disease resistance that is tightly associated with optimal levels of GSH. *N. tabacum* cv. Samsun *nn* plants treated with nutrient solutions containing either sufficient sulfate (+S) or no sulfate (−S) were evaluated during compatible interactions to TMV. Sufficient sulfate supply (+S) of tobacco elevated Cys and GSH contents and induced TMV resistance in these genetically susceptible plants as manifested by delayed mosaic symptoms and reduced virus accumulation, as compared to −S plants [86]. The same phenomenon was observed in genetically resistant tobacco (*N. tabacum* cv. Samsun *NN*), as sufficient sulfate supply (+S) resulted in the development of significantly less necrotic lesions and reduced TMV accumulation during an HR, as compared to plants grown without sulfate (−S) [87]. The identification of various GSH-deficient mutants of *A. thaliana* also demonstrated that adequate levels of GSH are important for the establishment of disease resistance. *Arabidopsis pad2-1* mutants displayed enhanced susceptibility to *P. syringae* pv. *maculicola* ES4326 (*Psm* ES4326) and the oomycete pathogen *Phytophthora brassicae*. It has been shown that *PAD2* encodes GSH1, a key enzyme of GSH biosynthesis [88]. Genetic complementation of GSH deficiency of *pad2-1* by overexpression of the wild-type *GSH1* cDNA was successful, since GSH levels and pathogen resistance were restored [88]. Notably, in *Arabidopsis pad2-1* mutants, GSH levels were reduced to 22% of those in wild-type plants and accompanied by a significant increase in Cys levels. It may seem contradictory that high levels of Cys did not induce resistance to *Psm* ES4326 [88], since in a different study, an increase in Cys levels did induce resistance in *Arabidopsis* to *Pst* DC3000 (see [21], discussed above). Álvarez et al., [21] used *DES1* knockout mutants of *A. thaliana*. DES1 uses Cys to produce H_2_S, so if DES1 does not function properly, Cys accumulates in the cytosol. Cys accumulation in *DES1* mutants was relatively marginal, only 1.5-fold compared to the wild-type control but it was sufficient to induce resistance to *Pst* DC3000. However, Parisy et al., [88] used *pad2-1* mutants deficient in GSH1, a key enzyme of GSH biosynthesis resulting in Cys contents 5-fold higher than wild type levels, a possible cause of the absence of resistance to *Psm* ES4326 besides GSH-deficiency.

### 3.3. GSH and Plant Hormones

GSH has been shown to modulate the defense signaling network by cross-communication with several biotic stress related phytohormones [89]. GSH regulates salicylic acid (SA) accumulation and plant resistance to different biotrophic pathogens via an SA-mediated pathway [90]. It has also been demonstrated that GSH induces ethylene (ET) and jasmonic acid (JA) as well. In a nutshell, we recapitulate here how GSH regulates these plant hormones during plant–pathogen interactions.

#### 3.3.1. GSH and SA

**Figure 2:**
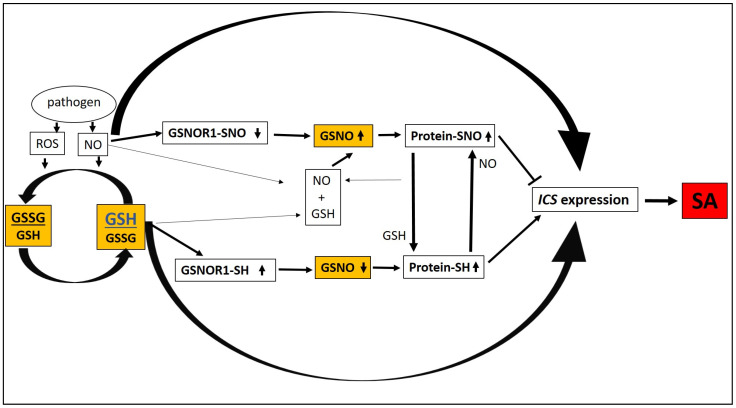
Pathogen induced defense signaling enhances the accumulation of the plant hormone salicylic acid (SA) through the expression of *isochorismate synthase* (*ICS*) and glutathione (reduced/oxidized form, GSH/GSSG) regulates this process in different ways. Reactive oxygen species (ROS) and nitrogen oxide (NO) formation during plant defense modulate the GSH/GSSG ratio and ultimately increase GSH levels in resistant plants. GSH and NO may positively modulate SA-dependent gene expression through *ICS*. GSH activates *S*-nitrosoglutathione reductase 1 (GSNOR1) that catalyzes the degradation of *S*-nitrosoglutathione (GSNO). GSNO degradation leads to a reduction in protein-SNO formation, therefore, protein-SH groups remain intact, activating enhanced *ICS* expression and SA synthesis. NO inactivates GSNOR1, leading to GSNO accumulation, protein-SNO formation and repression of *ICS* expression. In contrast, GSH can react with protein-SNOs to form protein -SH groups leading to enhanced *ICS* expression, SA accumulation and plant defense. Furthermore, not only the NO derived from the reduction in protein-SNOs but also NO accumulating during initial stages of plant defense to pathogens can react with GSH to form GSNO, which will repress SA accumulation and plant defense.

GSH has a complex role in SA-mediated defense responses. Signal molecules such as ROS and nitrogen monoxide (NO) play important roles in transmitting information during pathogen infections. ROS and NO accumulation is one of the earliest cellular responses following successful pathogen recognition [91,92,93,94,95]. Accumulation of one of the important ROS, hydrogen peroxide (H_2_O_2_) alters the GSH/GSSG ratio in *A. thaliana* and this change activates SA-associated plant defense signaling through the induction of the *isochorismate synthase 1* (*ICS1*) gene which encodes the key enzyme of SA biosynthesis in *Arabidopsis* [96]. Indeed, it has been shown that increasing GSH contents by overexpression of tomato *GSH1* in transgenic tobacco (*N. tabacum*) results in elevated GSH synthesis coupled to higher SA levels and these plants showed resistance to the bacterium *Pst* DC3000 [74]. *S*-nitrosoglutathione (GSNO) is an important *S*-nitrosylating agent in vivo that is formed by the reaction between NO and GSH [97]. GSNO induces SA biosynthesis through *ICS* and it is dependent on GSH. Moreover, NO regulates GSH biosynthesis and GSH/GSSG status of plant cells [98]. Concluding these results, NO and GSNO connect the ROS induced changes in GSH status to SA accumulation in plant cells. Furthermore, *S*-nitrosoglutathione reductase 1 (GSNOR1) regulates the level of GSNO in plant cells [99]. Loss of *AtGSNOR1* function increased protein-SNO levels in *A. thaliana*, disabling plant defense responses to *Pst* DC3000 and *Hyaloperonospora arabidopsidis* manifested as enhanced disease symptoms and pathogen reproduction. Conversely, increased AtGSNOR1 activity reduces protein-SNO formation and positively regulates the SA induced defense responses [99]. Others have recently shown that the activation of GSNOR1 enzyme leads to the release of inhibition of *ICS* expression in the presence of H_2_O_2_ [100]. However, when GSNOR1 is inactive, the accumulation of GSNO leads to the inhibition of *ICS* expression. Furthermore, the GSNOR enzyme is posttranslationally activated by direct denitrosylation in a GSH-dependent manner. Activation of *ICS* expression leads to SA accumulation [100]. In summary, the ROS and NO formation during plant defense modulate the GSH/GSSG ratio and ultimately increase GSH levels in resistant plants. Interactions between ROS, NO, GSH, GSNO and GSNOR lead to increased SA accumulation in different ways during incompatible plant-pathogen interactions (Figure 2). GSH cooperates with NO likely via unidentified (de)nitrosylation-dependent and independent pathways, to positively modulate SA-dependent gene expression such as that of *ICS1* [96,98,100]. The GSNOR enzyme controls plant GSNO levels and GSH activates GSNOR enzyme activity, which catalyzes GSNO degradation to GSSG and NH_3_ by using reduced β-nicotinamide adenine dinucleotide (NADH) in plant cells [101]. Decreasing GSNO levels leads to the reduction in protein-SNO formation therefore protein-SH mostly remains intact and this process activates enhanced *ICS* expression and SA accumulation. However, NO inactivates GSNOR, leading to the accumulation of GSNO, protein-SNO formation and the repression of *ICS* expression. On the other hand, GSH can react with protein-SNOs to form protein-SH leading to enhanced *ICS* expression, SA accumulation and plant defense. Furthermore, not only the NO derived from the reduction in protein-SNOs but also NO accumulating during initial stages of plant defense to pathogens can react with GSH to form GSNO, which will repress SA accumulation and plant defense (Figure 2).

**Figure 3:**
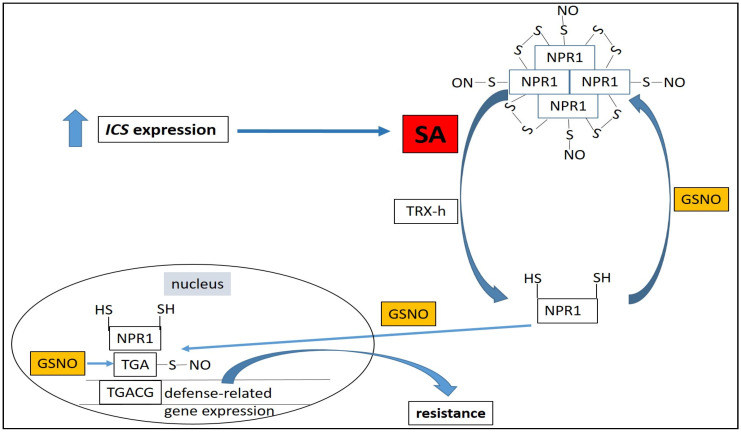
Salicylic acid (SA) accumulation induces defense gene expression through conformational changes of non-expressor of pathogenesis-related 1 protein (NPR1). During pathogen challenge changes in the redox status of plant cells leads to a reduction in cysteine residues in NPR1 and NPR1 monomers are released from the oligomeric, complex catalyzed by thioredoxins (TRX-h). In contrast, *S*-nitrosylation of NPR1 monomers by GSNO facilitates oligomerization. SA binding to the NPR1 oligomer is necessary for the final activation of monomerization. Activated NPR1 monomers are translocated from the cytoplasm to the nucleus mediated by GSNO. The activated NPR1 monomer induces *PR* expression in cooperation with TGA transcription factors and GSNO mediated *S*-nitrosylation of TGA enhances defense gene expression.

In unstressed plants SA synthesis is largely suppressed. We hypothesize that during the initial stages of infection, the elevation of GSH levels induced by the pathogen releases the suppression of SA accumulation. However, increased GSH levels will eventually elevate GSNO contents leading to suppression of SA accumulation which could be one possible mechanism of self-regulation of defense responses by the plant host. Within this complex multiplayer process described above, ROS, NO, GSH, GSNO and GSNOR work together to regulate SA levels, while pathogen-induced SA accumulation induces defense gene expression through conformational changes of non-expressor of pathogenesis-related 1 protein (NPR1). In unchallenged plants, NPR1 resides in the cytoplasm as an inactive oligomer maintained through redox-sensitive intermolecular disulfide bonds. *S*-nitrosylation of Cys156 residues of NPR1 is necessary for maintaining its oligomeric state. During pathogen challenge changes in the redox status of plant cells leads to the reduction in cysteine residues in NPR1 and NPR1 monomers are released from the oligomeric complex [102]. SA-induced NPR1 monomerization is catalyzed by thioredoxins (TRXs) via (1) a reduction in disulfide bridges between NPR1 molecules, (2) TRXh5 is also a direct protein-SNO reductase that can reduce S-nitrosylated Cys156 residues of NPR1 [103,104], while on the other hand, *S*-nitrosylation of NPR1 monomers by GSNO facilitates its oligomerization [103]. It was revealed later that an additional step is required for the SA-induced activation of NPR1. It has been shown that *Arabidopsis* NPR1 is an SA receptor and the binding of SA to NPR1 is necessary for the monomerization and final activation of NPR1 [105]. Activated monomers of NPR1 are then translocated from the cytoplasm to the nucleus [102,103] and GSNO treatment facilitates nuclear translocation and accumulation of NPR1 [98]. The activated NPR1 monomer induces *PR* expression in cooperation with TGA transcription factors in the nucleus. Interestingly, the GSNO mediated *S*-nitrosylation of TGA1 increased its DNA-binding activity in the presence of NPR1 [106]. Furthermore, GSNO treatments increased the expression of several *PR* genes (*PR-1*, *PR-2* and *PR-5*) and induced resistance to *Pst* DC3000 in *Arabidopsis* [98]. In summary: 1/ GSNO participates in the monomer-oligomer switch of NPR1, 2/ GSNO regulates the translocation of NPR1 monomer from the cytoplasm to the nucleus, 3/ GSNO activates TGA transcription factors in the nucleus and enhances the expression of *PR* genes in a GSH dependent manner. The interactions of GSNO in the defense responses downstream of SA are presented in (Figure 3).

Transgenic tobacco plants expressing the bacterial gene *NahG*, which encodes a salicylate hydroxylase, are unable to accumulate SA because the salicylate hydroxylase converts SA to cathecol [107,108]. Tobacco plants containing the *NahG* gene showed enhanced susceptibility to both virulent and avirulent pathogens [107,109]. We have demonstrated that increasing GSH levels in SA deficient tobacco (*N. tabacum* cv. Xanthi *NahG*), either by crossing with GSH overproducer transgenic tobacco lines or by injecting GSH or OTC into the leaves, maintains defense responses to TMV and to powdery mildew (*Euoidium longipes*) independently of SA accumulation [76,85].

#### 3.3.2. GSH and Jasmonic Acid

JA-dependent signaling has been reported to play a crucial role in pathogen attack, especially against necrotrophic pathogens. Necrotrophs, such as the bacterial pathogen *Erwinia carotovora* subsp. *atroseptica*, or the fungal pathogen *Alternaria brassicicola* kill host plant cells and acquire nutrients from dead or dying tissues inflicting devastating diseases and significant economic losses [110,111]. Interestingly, JA signaling has also been shown to mediate defense against hemibiotrophic pathogens such as *Xanthomonas oryzae* in rice [112]. In GSH deficient *cad2 Arabidopsis* mutants the expression of genes involved in JA synthesis and activation are altered as compared to wild-type plants [113]. Furthermore, these authors found that exogenous GSH treatments restore the JA-related defense gene expression in *cad2* mutants. In fact, JA-associated gene expression is induced by oxidative stress mediated by the GSH/GSSG status [113]. As we mentioned before, redox signaling by ROS and NO is crucial for SA signaling, however these redox changes, which lead to SA accumulation, are associated with the suppression of JA responses [114]. Indeed, *Arabidopsis* plants infected with necrotrophic *A. brassicicola* or *B. cinerea* showed increased plant defensin gene (*PDF1.2*) expression, which is a JA marker. However, when these plants were treated with SA, *PDF1.2* expression was reduced [115]. Furthermore, GSH was necessary for the suppression of *PDF1.2* in the presence of SA because the GSH biosynthesis inhibitor BSO strongly reduced the suppression of *PDF1.2*, suggesting that GSH induced redox modulation plays an important role in the SA-mediated attenuation of the JA signaling pathway [115].

#### 3.3.3. GSH and Ethylene

Ethylene (ET) is a gaseous phytohormone related to plant sulfur metabolism in different ways. Sulfur is necessary for ET biosynthesis because ET is synthetized in plants through *S*-adenosyl-l-methionine (SAM), the activated form of Met [116] (Figure 1). Furthermore, ET biosynthesis is regulated by GSH via SAM synthase (SAM1) [117], 1-aminocyclopropane-1-carboxylate synthase (ACS) and 1-aminocyclopropane-1-carboxylate oxidase (ACO) [75]. Transgenic *N. tabacum* plants overexpressing a tomato gene encoding a chloroplast-targeted GSH1 significantly upregulated ET biosynthesis genes (*ACS*, *ACO*) as compared to wild-type plants [75]. These GSH overproducer plants also showed increased SA accumulation, marked by enhanced *PR-1a* expression. The authors demonstrated that the increase in GSH contents is manifested by increased pathogen resistance to both the necrotrophic *B. cinerea* and the biotrophic *P. syringae* pv. *tabaci*, suggesting that GSH synergistically activates both SA and ET elevations [75]. In addition, transgenic *A. thaliana* plants overexpressing *GSH1* showed elevated GSH contents and improved resistance to the necrotrophic fungus *B. cinerea* [118]. These plants exhibited a strong upregulation of ET biosynthesis transcripts (ACS, ACO) while these genes were downregulated in the GSH-depleted *pad2-1* mutant. Furthermore, the ACO protein was post-translationally regulated by *S*-glutathionylation. These results clearly demonstrated that GSH-mediated resistance to necrotrophic plant pathogens may occur via an ethylene-mediated pathway [118].

### 3.4. Glutathione S-Transferases

Plant glutathione *S*-transferases (GSTs) are ubiquitous and multifunctional enzymes catalyzing the conjugation of GSH with endogenous and exogenous electrophilic compounds. GSTs participate in plant detoxification, as well as defense reactions to biotic stresses [119]. Certain plant GST isoenzymes have antioxidant (i.e., glutathione peroxidase) activity as well, since they catalyze the breakdown of lipid hydroperoxides derived from lipid peroxidation processes that occur, e.g., in dying plant cells. For example, *ShGST* is rapidly upregulated in resistant wild tomato plants (*Solanum habrochiates*) infected with a powdery mildew pathogen (*Oidium neolycopersici*), as compared to the susceptible *S. lycopersicum* cv. Mill. Silencing *ShGST* abolished the resistance to this biotrophic pathogen [120]. Furthermore, it has been described that smut disease caused by the biotroph *Sporisorium scitamineum* induces an early modulation of the production and scavenging of ROS during defense responses in resistant sugarcane. Pathogen spore germination and appressorium formation coincided with ROS accumulation in resistant plants, coupled with a reduced rate of lipid peroxidation and increased GST activities already at 12 h post inoculation [121]. It has been also shown that silencing of *GSTF9* in cotton (*Gossypium hirsutum*) resulted in enhanced susceptibility to *Verticillium dahliae* infection, as compared to wild-type plants [122], while transgenic *Arabidopsis* plants overexpressing *GaGSTF9* showed enhanced resistance [122]. Recently different GSTs have been identified as critical components of the glucosinolate and phytoalexin pathways [123,124], discussed below in detail. In summary, probably the most important function of GSTs in influencing the outcome of plant–pathogen interactions is the suppression of oxidative stress in infected host tissues via the contribution of GSH (see, e.g., [119]).

## 4. Sulfur Containing Pathogenesis Related (PR) Antimicrobial Peptides (AMPs) in Plant Disease Resistance

Plants have developed complex defense mechanisms to protect themselves against different pathogens. Pathogenesis-related proteins (PRs) are key elements of these mechanisms [125]. PRs have been classified into 17 families based on their biochemical and biological properties, and the well-characterized antimicrobial peptides (AMPs) such as defensins and thionins are classified into the PR-12 and PR-13 families, respectively [125]. Thionins and defensins are small (ranging from 5 to 7 kDa), usually basic, cysteine-rich peptides containing six to eight conserved cysteine residues. Based on their structure, thionins have been characterized as α/β-thionins and γ-thionins, the latter of which now we call defensins [126]. It has been predicted that more than 300 defensin-like genes may exist in *Arabidopsis* [127]. In general, AMPs are non-toxic to plant cells, however, they are extremely effective against bacterial or fungal pathogens. The main characteristic of AMPs is their broad in vitro antiviral, antifungal and antibacterial activity at micromolar concentrations [128,129,130]. AMPs have different modes of action against pathogens in vitro [131]. Plant defensins target various lipids of fungal membranes, such as sphingolipids and phospholipids [132,133]. After target interaction at the fungal plasma membrane, most but not all plant defensins are taken up by the fungal cell. The mechanisms of defensin-elicited fungal cell death can differ as well, including membrane permeabilization [134], overproduction of ROS in fungal cells [135], defensin induced apoptosis [136], cell lysis immediately after defensin exposure [133].

It has been found that *Arabidopsis* contains two genes that encode highly homologous plant defensins having totally different expression patterns. The defensin *PDF1.1* is expressed in seeds constitutively, whereas *PDF1.2* is expressed in leaves upon pathogen challenge with *Alternaria brassicicola* and shows antifungal activity in vitro [137]. Furthermore, they found that ROS producing agents (paraquat, rose bengal) or plant hormones such as ET and methyl JA induce *PDF1.2*, however, SA or 2,6-dichloroisonicotinic acid (INA), a synthetic SA analog cannot. Moreover, in SA-deficient (*NahG*) *Arabidopsis PDF1.2* expression is not inhibited in the absence of SA, therefore, the authors concluded that *PDF1.2* expression is independent of the SA-mediated defense pathway [137]. Plants exhibit a durable resistance, called non-host resistance, against non-adapted pathogens and it has been reported that induced expression of multiple plant defensins in *Arabidopsis* during non-host resistance is critical to prevent the infection of the non-adapted *Colletotrichum gloeosporioides* pathogen [138]. The induced expression of plant defensins in response to pathogen attack is mediated by the enhanced disease resistance1 (EDR1) protein kinase in *Arabidopsis* through the derepression of the transcription factor, MYC2, which regulates JA-responsive pathogen defense genes such as defensins [138]. In fact, these results are in line with the earlier findings of Penninckx et al. [137] showing that plant defensin induction is regulated by JA rather than SA. Furthermore, it was found that EDR1 is also involved in limiting the pathogenesis of host-adapted pathogens such as *A. brassicicola* and *C. higginsianum*, indicating that the EDR1 pathway contributes to both non-host resistance and basal defense responses through the derepression of defensin gene expression in response to pathogen attack [138]. It has been reported for the first time that a plant defensin is also effective against an obligate biotrophic pathogen (*Phakopsora pachyrhizi*), which causes Asian soybean rust [139]. The authors showed that recombinant pea defensin Drr230a inhibited spore germination in vitro and in planta to prevent infection by the non-adapted *P. pachyrhizi*. Furthermore, Drr230a significantly reduced disease symptoms and uredospore development in soybean leaflets [139]. Furthermore, it has been presented that a unique bi-domain defensin (MtDef_5_) from *Medicago truncatula* presents antibacterial activity and is effective against the plant pathogen *Xanthomonas campestris* pv. *campestris* [140]. MtDef_5_ is larger than normal defensins, contains 107 amino acids and is separated into two domains, MtDef_5_A and MtDef_5_B, 50 amino acids each, linked by a short peptide, APKKVEP. Interestingly, the single domain MtDef_5_B exhibits more potent antibacterial activity against *X. campestris* than MtDef_5_ in vitro. MtDef_5_, MtDef_5_A and MtDef_5_B increased bacterial cell membrane permeability, furthermore, MtDef_5_ and MtDef_5_B translocated through the bacterial cell membrane and accumulated in the *X. campestris* cytoplasm, subsequently binding to bacterial DNA [140].

Expression of different AMPs in transgenic plants successfully increases disease resistance against a broad range of pathogens [141]. Banana (*Musa* spp.), one of the most important food crops in the world, overexpressing *Petunia* floral defensin genes (*PhDef1* and *PhDef2*) showed enhanced resistance to *Fusarium oxysporum* f. sp. *cubense* and *Mycosphaerella fijiensis* [142]. Others have shown that the secreted antifungal protein thionin 2.4 (Thi2.4) in *A. thaliana* has a dual role in defense against *Fusarium graminearum* [143]. Transgenic Thi2.4 overexpressor *Arabidopsis* showed increased resistance to *F. graminearum* compared to wild type plants. Furthermore, it was found that Thi2.4 proteins are released to the extracellular space and interact with fungal fruit body lectin (FFBL) of *F. graminearum*. FFBL is toxic to *Arabidopsis* cells and Thi2.4 suppresses FFBL toxicity. Overall, Thi2.4 has antifungal activity and it is also able to suppress FFBL toxicity [143]. Another similar example is a cold induced defensin (TAD1) present in winter wheat (*Triticum aestivum*) that confers in vitro resistance to the snow mold pathogen *Typhula ishikariensis.* In fact, the low temperature during overwintering was necessary in inducing resistance to snow mold [144]. Furthermore, transgenic wheat plants overexpressing *TAD1* show increased resistance not only against *T. ishikariensis* but also to *F. graminearum* [144]. It has been presented recently that transgenic *Arabidopsis* plants expressing a modified thionin (*Mthionin*) also showed reduced *Fusarium graminearum* development by inhibiting fungal spore germination and hyphal growth in planta [145]. This study demonstrated that Mthionin may enhance SA/JA-mediated defense against *F. graminearum* infection. However, *Mthionin* expression in transgenic *Arabidopsis* did not affect the plant microbiome [145]. In summary, it seems that in general plant AMPs, these sulfur (cysteine) rich peptides can specifically limit infection by a given pathogen in a particular host(s) without exerting a significant influence on the host microbiome. The mode of action of AMPs is well characterized in vitro, however, further experiments are necessary to reveal the exact role of AMPs during pathogen attack. It seems that plant hormones are the main signaling molecules in the activation of AMPs in disease resistant plants.

## 5. Sulfur-Containing Secondary Metabolites (Phytoalexins, Phytoanticipins) in Plant Disease Resistance

Sulfur-containing secondary metabolites play an important role in plant disease resistance and these defense compounds based on their mode of actions can be classified into phytoalexins and phytoanticipins [146,147]. Phytoalexins are only synthesized in plants after pathogen infection (or herbivore attack) and it requires de novo gene expression and the production of enzymes leading to the installation of new biosynthetic pathways not usually present in the unchallenged plant [148]. In contrast, phytoanticipins are already in place before any external attack by pathogens, or are synthesized immediately from inactive precursors already present in the plants with no expenditure of cellular energy [147].

### 5.1. Sulfur-Containing Phytoalexins

Phytoalexins are highly diverse, low molecular weight antimicrobial compounds that are produced in different plant species in response to pathogen infection. *Brassicaceae* plants produce phytoalexins which are usually composed of an indole core and a side chain with one or two sulfur atoms [149]. This review only deals with sulfur-containing indole-type phytoalexins such as camalexin, brassinin and rapalexin A. Among these compounds a contribution to plant defense in vivo has only been proven for camalexin [150]. Other *Brassicaceae* phytoalexins are also postulated to be critical for plant immunity. However, their antimicrobial properties have been revealed only during in vitro assays with a range of different pathogens [149]. Their contribution to plant resistance is also indicated by the fact that plant pathogenic fungi attempt to detoxify different phytoalexins during infection (see [151] and references within).

**Figure 4:**
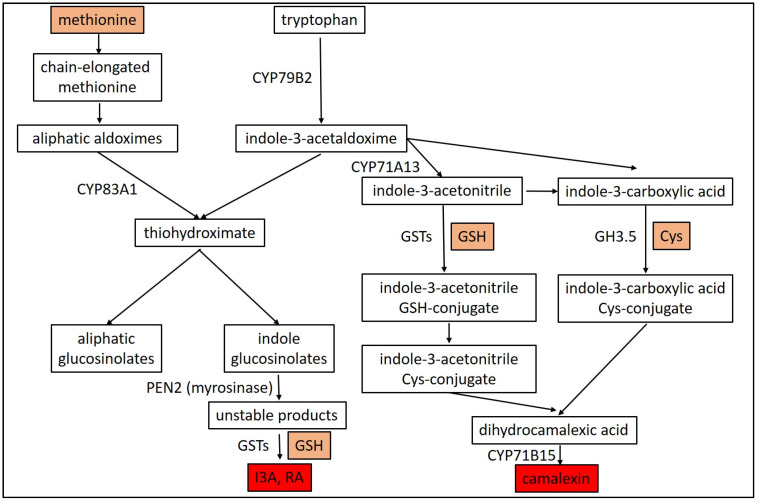
Glutathione (GSH) and cysteine (Cys) are involved in the in planta biosynthesis of camalexin and indol glucosinolates, compounds that contribute to resistance to fungal infections. CYP79B2, CYP71A13 and CYP71B15 = cytochrome P450 enzymes required for camalexin biosynthesis from tryptophan in *Arabidopsis thaliana*; CYP83A1 = a cytochrome P450 monooxygenase responsible for the aliphatic glucosinolate pathway; GSTs = glutathione-S- transferases; GH3.5 = acetyl-amido synthetase; I3A, RA = end products of PEN2-mediated indol glucosinolate hydrolysis. For further explanations and details see the text.

In sulfur-deficient plants, there is a general down-regulation of genes responsible for synthesis of sulfur containing secondary metabolites and therefore camalexin biosynthesis is also inhibited. On the other hand, sulfur deficiency is also accompanied by an up-regulation of genes controlling sulfur uptake and assimilation [152]. In contrast, the formation of camalexin is enhanced in *A. thaliana* infected with *Alternaria brassicicola* grown with an optimal, as compared to a suboptimal sulfate supply [8]. Sulfur deprived plants show reduced levels of GSH [86], since GSH functions as a molecule that provides reduced sulfur to other sulfur-containing secondary metabolites, such as camalexin. Therefore, camalexin levels are also reduced in GSH deprived plants [88,153]. As mentioned before, *PAD2* encodes GSH1, a key enzyme in GSH biosynthesis [88]. Phytoalexin deficient *Arabidopsis* mutants (*pad2-1*) showed reduced levels of GSH and camalexin, coupled to an enhanced susceptibility to bacterial infections [88]. Reduced accumulation of camalexin in *pad2-1* mutant plants suggests that GSH is the precursor to the thiazole ring of camalexine [88]. Camalexin is synthesized from tryptophan through indole-3-acetonitrile (IAN), and IAN then conjugates with GSH to form GS-IAN [154]. Different GSTs (GSTF6, GSTU4) are probably involved in camalexin biosynthesis by catalyzing the GS-IAN conjugation [123,124,155] (Figure 4).

Furthermore, an alternative camalexin biosynthesis pathway was demonstrated showing that the multifunctional acetyl-amido synthetase GH3.5 enzyme in *Arabidopsis* is involved in camalexin biosynthesis via conjugating indole-3-carboxylic acid and Cys [156] (Figure 4). Camalexin biosynthesis from tryptophan requires several cytochrome P450 enzymes, including CYP79B2, CYP71A13, and CYP71B15 [157]. It has been shown that *PAD3* encodes the multifunctional cytochrome P450 enzyme CYP71B15 which catalyzes the final step of camalexin biosynthesis in *Arabidopsis* [158]. Indeed, in phytoalexin deficient *Arabidopsis pad3* mutants the lack of camalexin leads to enhanced susceptibility to different pathogens such as *A. brassicicola* [159], *B. cinerea* [160] and *Leptosphaeria maculans* [161]. Interestingly, however, an *Arabidopsis cyp83a1-3* mutant was identified, which shows enhanced resistance to the powdery mildew fungus *Golovinomyces cichoracearum* coupled to increased camalexin accumulation [162]. These authors showed that wild type *Cyp83a1-3* encodes a cytochrome P450 83A1 monooxygenase (CYP83A1) [162]. Interestingly, when the aliphatic glucosinolate pathway is blocked because of the *cyp83a1* mutation, the pathway for indole-derived products, including IGSLs and camalexin, is enhanced [158,162,163] (Figure 4). In addition, overexpression of *PAD3* in *Arabidopsis* leads to enhanced camalexin accumulation and increased *G. cichoracearum* resistance that is comparable to the disease resistance of *cyp83a1-3* mutants [162]. Several reports have shown that camalexin biosynthesis is regulated through MAPK cascades [148]. For example, it has been presented that the biosynthesis of camalexin, in *Arabidopsis* is regulated by the MPK3/MPK6 cascade in response to *Botrytis cinerea* [164]. It has been observed that during *B. cinerea* spore germination the activation of MPK3 and MPK6 is induced in *Arabidopsis* seedlings, followed by accumulation of camalexin, while camalexin accumulation is reduced in *mpk3* and delayed in *mpk6* mutants. Importantly, in the double mutant *mpk3/mpk6* the induction of camalexin is almost abolished, demonstrating that both MPK3 and MPK6 are involved in fungus-induced camalexin production [164]. Others have found that the phosphorylation of the WRKY33 transcription factor is required for MPK3/MPK6-induced camalexin biosynthesis in response to *B. cinerea* infection [165]. Because camalexin and other phytoalexins are toxic to the plant, specific transporters are needed for their secretion. *Arabidopsis thaliana* produce and secrete camalexin in response to *Alternaria brassicicola* infection and an ATP-binding cassette transporter (ABCG34) mediates the secretion of camalexin from epidermal cells to the leaves surface, conferring thereby resistance to *A. brassicicola* infection [166]. *Arabidopsis* plants overexpressing *AtABCG34* secreted more camalexin to the leaf surface and showed an enhanced defense response to the pathogen, whereas *atabcg34* mutants secreted less camalexin and showed enhanced susceptibility to *A. brassicicola* [166].

Elemental sulfur (S^0^), which is the oldest pesticide used by mankind, is interestingly also produced by various plant species such as cocoa [167], tomato [168], tobacco, cotton and French beans [169]. S^0^ can be regarded as the only inorganic phytoalexin in plants that accumulates during the infection of xylem-invading fungal and bacterial pathogens and its accumulation is faster and greater in disease resistant genotypes then in susceptible lines [170]. A positive correlation has been shown between S^0^ accumulation and decreased hyphae colonization by *Verticilium dahliae* in infected tomatoes [168]. However, the in planta biosynthesis of S^0^ and its mode of action during pathogen infections is still unknown.

### 5.2. Phytoanticipins

#### 5.2.1. Glucosinolates

Glucosinolates (GSLs) are sulfur-rich secondary metabolites with antimicrobial activity found specifically in the *Brassicales* order which includes important crops such as oilseed rape (*Brassica napus*), cabbage (*B. oleracea* var. *capitata*), broccoli (*B. oleracea* var. *italica*), turnip (*B. rapa* subsp. *rapa*), white mustard (*Sinapis alba*), as well as the model plant *A. thaliana* [28]. GSLs are constitutively produced defense metabolites that are synthesized independently of a pathogen attack, but they are activated by mirosinase enzymes (β-thioglycoside glucohydrolases) during infection, whereas phytoalexins are formed in response to the pathogen infections [171]. GSLs share a chemical structure consisting of a β-d-glucopyranose residue linked via a sulfur atom to a (*Z*)-*N*-hydroximinosulfate ester, plus a variable R group derived from amino acids. Based on the precursor amino acid, GSLs can be classified into aliphatic glucosinolates, aromatic glucosinolates, and indole glucosinolates (iGSLs) [172]. GSL contents may be affected by the sulfur nutritional status of the plant; supplemental sulfur fertilization of *Brassica* in greenhouse and field experiments resulted in an up to 20-fold increase in GSL contents in foliar tissues [152]. Furthermore, it has been found that a seven-day sulfate deprivation significantly reduced GSL contents in *Brassica juncea* and *B. rapa* [173]. In unstressed plants GSLs are stored in laticifer-like S-cells within the phloem cap region [174] and within plant seeds [175]. Interestingly, seeds are unable to de novo synthesize GSLs, therefore, GSL transporters and importers are necessary for loading GSLs into seeds during maturation [175]. GSLs are relatively non-reactive compounds, however, during pathogen infection GSLs are rapidly hydrolyzed by myrosinases to produce different physiologically active toxic compounds such as isothiocyanates, thiocyanates, nitriles and epithionitriles [124,176,177]. The production of various end products of GSLs are organ-specifically regulated in *A. thaliana*, including the production of nitriles in roots, at the expense of isothiocyanates in rosette leaves [178]. Furthermore, it has been found that appropriate GSH levels are important for the execution of plant defense mechanisms in response to pathogens mediated by PENETRATION2 (PEN2) myrosinase [124]. This enzyme hydrolyzes GSLs in response to attempts of pathogenic infections. PEN2-mediated GSL hydrolysis leads to the formation of several end products including indol-3-yl methyl amine (I3A), raphanusamic acid (RA), and 4-*O*-β-d-glucosyl-indol-3-yl formamide [179,180,181]. In GSH-deficient plants a reduced accumulation of I3A and RA has been observed, suggesting a contribution of GSH to PEN2-mediated GSL hydrolysis during plant disease resistance. In fact, this defense pathway involves conjugation of GSH with unstable products of GSL metabolism and further processing of the resulting adducts to biologically active molecules mediated by GSTU13 [124]. It has been shown that a lack of functional GSTU13 in *Arabidopsis* results in enhanced disease susceptibility toward several fungal pathogens (*Erysiphe pisi*, *Colletotrichum gloeosporioides*, and *Plectosphaerella cucumerina*) [124]. GSLs have a huge impact on plant disease resistance, however, the signaling processes leading to GSL accumulation and conversion to toxic products have been elusive. Recently, it has been revealed that the MPK3/MPK6 MAP kinase cascade regulates indole-3-yl-methylglucosinolate biosynthesis and its conversion to 4-methoxyindole-3-yl-methylglucosinolate in response to the necrotrophic pathogen *Botrytis cinerea* [176]. Targeted delivery of toxic antimicrobial end products to pathogen contact sites is necessary for successful plant defense to attempted pathogenic infection. It has been shown recently that the phytoalexin camalexin and isothiocyanates which are hydrolysis products of GSLs are transported to the apoplast redundantly through PEN3 and PDR12 multifunctional transporters [182]. Accumulation of camalexin and isothiocyanates in the apoplast leads to the inhibition of *B. cinerea* [182]. The *Arabidopsis pen* (*pen1*, *pen2* and *pen3*) mutants were originally isolated as plants displaying loss of pre-penetration defense against the non-host pathogen *Blumeria graminis* f. sp. *hordei* (*Bgh*). During non-host interactions, *Bgh* typically fails to enter the attacked *Arabidopsis* cell. However, Arabidopsis *pen1* and *pen2* mutants infected with *Bgh* fail to block the entry of the non-host pathogen [183]. Later it was described that PEN2 limits growth of a wide spectrum of pathogens, whereas PEN1 function is limited to non-host powdery mildew species [183]. PEN1 encodes a plasma membrane-anchored syntaxin, a potential key player in vesicle-associated membrane fusion and secretion processes, including exocytosis [184]. The presence of a functional PEN1 homolog, ROR2 (REQUIRED FOR MLO RESISTANCE 2) in the monocot species barley suggests the existence of an evolutionarily ancient defense mechanism. In barley, pre-penetration defense to *Bgh* at sites of attempted pathogen ingress is associated with ROR2-mediated formation of vesicles that contain the ROS H_2_O_2_ [184]. It is likely that *Arabidopsis* PEN1 also confers H_2_O_2_ accumulation during defense to non-host powdery mildews, since non-host resistance of cowpea to *Erysiphe cichoracearum* is partially suppressed by exogenous application of catalase, promoting H_2_O_2_ degradation [185]. Later it has been described that PEN2 is a myrosinase [179] and PEN3 is a multifunctional transporter that transports toxic GSL end products to the apoplast [182], suggesting that functions of PEN1, PEN2 and PEN3 link GSL- and ROS-mediated plant disease resistance responses. The PEN2/PEN3-dependent extracellular defense contributes to *Arabidopsis* resistance against a variety of fungal and oomycete pathogens [179,182]. It has been demonstrated that in *Arabidopsis* iGSLs and the phytoalexin camalexin work together in order to prevent *Phytophthora brassicae* infection [186]. These authors showed an early accumulation (6 h after inoculation) of indole-type GSL degradation products through PEN2 myrosinase mediated hydrolysis which leads to an active penetration resistance during pathogen attack. Furthermore, they found that GSL hydrolysis and action occurred in the absence of cellular destruction. Moreover, camalexin accumulation restricts subsequent pathogen development and further spread to neighboring cells [186]. It has been shown that *Arabidopsis* plants overexpressing the myrosinase β-*thioglycoside glucohydrolase 1* (*BoTGG1*) gene from broccoli (*Brassica oleracea* var. *italica*) show enhanced resistance to the bacterial pathogen *Pst* DC3000 [187]. Overexpression of *BoTGG1* in *A. thaliana* leads to accelerated stomatal closure and inhibited stomatal reopening during the infection of *Pst* DC3000 [187]. Later it was described that the host response to *Verticilium longisporum* infection differs in *Brassica napus* plants grown in sulfur sufficient vs. deficient conditions [188]. These authors found that infected plants always showed higher contents of sulfur-containing metabolites, such as specific GSLs, in comparison to non-infected plants. Sufficient sulfur fertilization is generally reflected in higher contents of sulfur-containing compounds, as well as a lower rate of infection compared to sulfur-deprived plants [188]. Remarkably, they showed that infection with *V. longisporum* also seems to enhance the synthesis of iGSLs in sulfur-deprived *B. napus* plants; despite the fact that these plants are very deficient in sulfur they managed to synthesize more iGSLs compared to their infected sulfur-sufficient counterparts. This phenomenon highlights the importance of iGSLs in plant defense [188].

#### 5.2.2. Thiosulfinates

Besides GSLs one of the most important phytoanticipins are thiosulfinates, which are produced in high amounts by e.g., *Allium* species. The diallylthiosulfinate allicin is a volatile, organosulfur, prooxidant compound from garlic (*Allium sativum*) with a broad spectrum of biological activities. Allicin is produced upon tissue damage from alliin (*S*-allyl-l-cysteine sulfoxide), a non-proteinogenic amino acid in a reaction catalyzed by the enzyme alliinase [189]. The proposed biosynthetic pathway of alliin is originated from GSH through different catalytic steps [190]. Allicin is able to oxidize cysteine residues of GSH and proteins. Oxidation of protein Cys-SH residues can lead to changes in protein structure, which affect the functions of the protein (see [104] and references within). Thiosulfinates have also been demonstrated to be effective against garlic pests. A positive correlation was detected between thiosulfinate contents and resistance to the underground pest *Bradysia odoriphaga* [191]. Remarkably, it seems that thiosulfinates also play a role in resistance to pathogens. A strong association between the genetic requirements for the bacterium *Pantoea ananatis* to colonize necrotized onion tissues and its capacity for tolerance to the thiosulfinate allicin has been found based on the presence of an eleven-gene, plasmid-borne, virulence cluster of sulfur redox genes in the bacterial genome [192]. Furthermore, genomic clones from a highly allicin-tolerant bacterium *Pseudomonas fluorescens* isolated from garlic conferred allicin tolerance to *Pseudomonas syringae* [193]. In addition, methyl methanethiolsulfinates, the hydrolysis products of *S*-Methyl-l-cysteine sulfoxide, have been shown to be important antibacterial compounds in cabbage effective against *Leuconostoc mesenteroides* [194,195].

## 6. Reactive Sulfur Species (RSS)

Among the various defense-related sulfur compounds, reactive sulfur species (RSS) are currently in the focus of interest of numerous research groups due to their participation in cellular signaling and regulatory processes. RSS are a diverse group of redox active sulfur containing compounds that are capable of either oxidizing or reducing biomolecules under physiological conditions. RSS include among others H_2_S, sulfenic acid, sulfinic acid, thiyl radicals, thiosulfinates, thiosulfonates, various persulfides and polysulfides [196,197,198]. Sulfur has unique chemical properties because it occurs in a wide range of oxidation states (from −2 to +6) in different compounds, and hence sulfur-derived metabolites are major participants of redox metabolism and post-translational modifications as well as of detoxification processes. Two RSSs, hydrogen sulfide and sodium sulfite have been recently shown to play important roles in plant disease resistance [7,199,200,201,202,203].

Hydrogen sulfide (H_2_S) is a highly reactive and toxic molecule that has recently emerged as an important signaling compound with many physiological functions in both health and disease. In mammalian systems, the possible role of H_2_S as an endogenous neuromodulator was first described in 1996, and the molecule is now accepted as the third most prevalent gasotransmitter after nitric oxide (NO) and carbon monoxide (CO) [204]. In plants, H_2_S also functions as an important signaling molecule, similar to NO or H_2_O_2_ [7,31,205,206]. Various l-cysteine desulfhydrase enzymes are involved in the degradation of cysteine and are therefore responsible for the generation of H_2_S [21,31]. Recent studies have suggested that not H_2_S, but rather H_2_S donor sulfane sulfur compounds act as signaling molecules and are responsible for the biological activities of some RSS [207]. H_2_S influences several physiological processes, it promotes root organogenesis, seed germination, lateral root formation and enhances photosynthesis [201,203]. H_2_S treatment of plants confers protective roles in responses to heat, drought, salt, osmotic, and freezing stresses [203,205].

In plants, H_2_S plays important roles in disease resistance but the underlying mechanisms are still largely unknown [7,208,209]. The most often proposed mechanism is the post-translational modification of redox-sensitive cysteine residues in various proteins. This process is called *S*-sulfhydration or persulfidation, which means the conversion of cysteine sulfhydryl groups to persulfide (-SSH) residues that can profoundly affect the function of various enzymes containing pivotal cysteine residues in their active centers [210]. By applying sodium sulfide (Na_2_S) treatments numerous proteins were post-translationally modified via *S*-sulfhydration in *A. thaliana* under physiological conditions. The sulfide added through *S*-sulfhydration reversibly regulated the activities of plant proteins in a manner similar to that described in mammalian systems [210,211].

A strong increase in H_2_S emission was observed in oilseed rape (*B. napus*) following fungal infection with *Sclerotinia sclerotiorum* [208]. *A. thaliana* plants treated with sodium hydrosulfide (NaHS, which is a H_2_S donor) exhibited improved resistance against *Pseudomonas syringae* pv. tomato DC3000. The transcript levels of the defense genes *Enhanced Disease Susceptibility 1* (*EDS1*), *Phytoalexin Deficient* 4 (*PAD4*), *PR1*, *PR2*, *PR3*, *PR4*, and *PR5* were also up-regulated in NaHS-treated plants [203]. H_2_S markedly increased the abundance of several defense-related proteins also in spinach leaves [201]. Moreover, H_2_S inhibited the accumulation of reactive oxygen species (ROS) and regulated the cellular content of 50 metabolites including amino acids, organic acids, sugars, sugar alcohols, and aromatic amines. Taken together, these results indicated that l-cysteine desulfhydrase and H_2_S conferred biotic stress resistance, via affecting stress-related gene expression, ROS metabolism and metabolic homeostasis [203]. Interestingly, WRKY transcription factor proteins were shown to regulate the expression of several genes participating in H_2_S biosynthesis [202]. H_2_S released by NaHS treatment effectively reduced the postharvest decay of fruits induced by *Aspergillus niger* and *Penicillium italicum*. Furthermore, H_2_S inhibited spore germination, germ tube elongation, mycelial growth, and produced abnormal mycelial contractions under in vitro conditions [36].

Importantly, the metabolism of H_2_S is closely connected to those of important plant defense hormones such as SA, JA and ET. H_2_S was shown to act as a downstream signal molecule in SA-induced heat-tolerance of maize seedlings [212]. SA treatment enhanced the activity of l-cysteine desulfhydrase, which in turn induced a marked accumulation of endogenous H_2_S. Interestingly, the SA-induced heat tolerance was enhanced by addition of NaHS, but weakened by specific inhibitors of H_2_S biosynthesis [212]. The signaling network of JA is also associated with H_2_S. JA could enhance the generation of endogenous H_2_S and l-cysteine desulfhydrase activity in guard cells of *Vicia faba* leaves. H_2_S may function downstream of H_2_O_2_ in JA-induced stomatal closure [213]. In addition, the proteomic analysis of H_2_S-treated spinach leaves revealed a markedly increased abundance of lipoxygenase (LOX) proteins [201]. LOX enzymes are known to participate in JA biosynthetic pathways [214]. On the other hand, Cys and GSH contents and biosynthesis are regulated by JA at the transcriptional level [35]. The metabolism of Cys is linked to ET biosynthesis [215,216]. Exogenous application of ET could significantly increase endogenous H_2_S content in *Arabidopsis* seedlings [217]. On the other hand, ET biosynthesis is associated with H_2_S signaling [201].

Besides H_2_S, the metabolism of another RSS, the sulfite anion has recently been also associated with plant disease resistance as a possible signal molecule [200,218,219]. Endogenous sulfite (SO_3_^2−^) levels in *A. thaliana* and tomato were determined by Brychkova [220]. Sulfite above a threshold level is toxic and it is rapidly metabolized in plants [221]. In the sulfur assimilation pathway sulfite is reduced by sulfite reductase by a process that transfers six electrons from ferredoxin to produce the fully reduced sulfide form for incorporation into cysteine [221]. Alternatively, sulfite can also be oxidized to sulfate by sulfite oxidase, which is a molybdenum cofactor-containing enzyme localized in peroxisomes [222,223]. Interestingly, the genes encoding sulfite oxidase, sulfite reductase and adenosine 5′-phosphosulfate kinase enzymes were markedly up-regulated in *Hibiscus chlorotic ringspot virus* (HCRSV)-infected plant leaves. The up-regulation of the sulfite oxidase gene was related to suppression of symptom development induced by sulfur treatment [199,224].

The gaseous pollutant sulfur dioxide (SO_2_) readily hydrates in plants at apoplastic pH to form the sulfite ions HSO_3_^1−^ and SO_3_^2−^, which are strong nucleophiles that can deleteriously react with a wide variety of cellular components [222]. Transcriptome analysis carried out on grape berries treated with SO_2_ revealed a broad perturbation of redox poise and a large-scale stress response. Sulfite was directed towards chelation and conjugation and uncoupled from oxidation to sulfate. Accordingly, numerous genes encoding GSTs were up-regulated along with a down-regulation of components involved in redox homeostasis. Tolerance and defense mechanisms were up-regulated, notably signaling via auxin, ET and JA [200]. Numerous genes encoding pathogenesis-related proteins and enzymes required for the phenylpropanoid pathway and for cell wall modification were highly activated in *A. thaliana* upon SO_2_ exposure [218]. Transcriptome-wide analysis of *A. thaliana* plants fumigated with SO_2_ revealed that large amounts of sulfite were involved in sulfur assimilatory pathways directly and uncoupled from sulfite oxidative pathways. Furthermore, transcripts associated with biotic stress, as well as with reactive oxygen species generating and scavenging pathways were markedly up-regulated [219]. Interestingly, pre-treatment of *A. thaliana* plants with SO_2_ also resulted in significantly enhanced resistance to infection with the necrotrophic fungus *B. cinerea*. SO_2_ pre-treatment markedly enhanced the activities of defense-related enzymes including phenylalanine ammonia-lyase (PAL), polyphenol oxidase, and PR-proteins. Additionally, the miRNA-mediated suppression of the auxin signaling pathway was observed [225]. In addition, SO_2_ application during postharvest storage successfully inhibited the development of *B. cinerea* in grape berries [226]. Importantly, these authors demonstrated that SO_2_ treatment, beyond a direct antifungal effect, also activated plant defense responses manifested as an enhanced expression of different grape *PR*-genes (*chitinase, β-1,3-glucanase*) and *PAL*, which encodes the key enzyme of the phenylpropanoid pathway [226].

## 7. Conclusions and Future Perspectives

Sufficient levels of sulfur in soils confer the optimal plant uptake of inorganic sulfate salts, a prerequisite for sulfur-containing defense compound (SDC) concentrations required for plant disease resistance responses. Indeed, sufficient sulfur fertilization is generally reflected in higher contents of SDCs, as well as a lower rate of infection compared to sulfur-deprived plants. In spite of the very diverse chemical structures of SDCs, there are some similarities in their modes of action against pathogens. SDCs are instrumental both in pathogen perception and initiating resistance-associated signal transduction pathways. Importantly, these processes are interconnected with various defense responses regulated by plant hormones (in particular, salicylic acid, jasmonic acid and ethylene), NO and reactive oxygen species (ROS). Sulfur-derived metabolites are major participants of plant redox metabolism and post-translational modifications as well as of detoxification processes. In fact, the unique chemical properties of sulfur (S), occurring in a wide range of oxidation states in various compounds, may contribute to the versatile roles of SDCs in plant resistance responses to pathogens. On the other hand, diverse S-containing compounds also have specific roles. An important characteristic of Cys is that it is the central hub of plant sulfur metabolism, in particular, Cys is a precursor molecule of numerous SDCs. Met and the Met cycle is connected to DNA, RNA and histone methylation reactions as well as to the biosynthesis of the plant hormone ethylene and polyamines. GSH participates in antioxidative and detoxification reactions and conducts the signaling of different plant hormones during pathogen infection. Importantly, a self-regulating circuit of H_2_O_2_, NO, glutathione and salicylic acid (SA) controls SA-mediated defense responses to bacterial and fungal infections [96,98,99,100]. Future research should clarify whether the same/similar self-regulating signaling is also responsible for the efficiency of SA-mediated plant defense responses during viral infections. Cysteine-rich peptides like defensins and thionins show direct antimicrobial effects and have additional roles in plant growth and development. Phytoanticipins are preformed SDCs which are already present before the plant is attacked, or which are produced rapidly and spontaneously from a preformed substrate by simple chemical or enzymatic modifications via a pre-existing enzyme. A unique characteristic of RSS is *S*-sulfhydration or persulfidation of redox-sensitive cysteine residues in various defense-associated proteins.

Successful plant defense against pathogen attack (i.e., resistance) is often associated with enhanced ROS production (oxidative burst). In this regard, the recent discovery of the first extracellular H_2_O_2_ receptor (HPCA1) in plants [43] provides a missing link to the in planta operation of so-called ROS waves. These ROS producing waves are initiated upon stress-exposure and confer a rapid, H_2_O_2_-mediated cell-to-cell defense signaling. ROS bursts ultimately result in different types of reversible oxidation (disulfide formation, sulfenylation, glutathionylation) of cysteine (Cys) residues on various plant proteins. These plant redox modifications (redoxome) have profound effects on multiple protein functions like catalytic activity, subcellular localization and, last but not least, the signal transduction of plant defense responses during pathogen infections. However, the impact of pathogen-triggered ROS bursts and, in particular, SDCs on the diverse oxidative cysteine modifications of plant proteins is still only partially characterized. The future engineering of these sulfur-associated redox-switches by, e.g., gene editing should enable a temporally and spatially targeted induction of defense responses of crops to a given pathogen.
